# Compatibility between Co-Metallized PbTe Thermoelectric Legs and an Ag–Cu–In Brazing Alloy

**DOI:** 10.3390/ma11010099

**Published:** 2018-01-10

**Authors:** Dana Ben-Ayoun, Yatir Sadia, Yaniv Gelbstein

**Affiliations:** 1 Unit of Energy Engineering, Ben-Gurion University of the Negev, Beer-Sheva 84105, Israel; yanivge@bgu.ac.il; 2 Department of Materials Engineering, Ben-Gurion University of the Negev, Beer-Sheva 84105, Israel; yatttir@yahoo.com

**Keywords:** thermoelectrics, PbTe, bonding, metallic contacts, brazing

## Abstract

In thermoelectric (TE) generators, maximizing the efficiency of conversion of direct heat to electricity requires the reduction of any thermal and electrical contact resistances between the TE legs and the metallic contacts. This requirement is especially challenging in the development of intermediate to high-temperature TE generators. PbTe-based TE materials are known to be highly efficient up to temperatures of around 500 °C; however, only a few practical TE generators based on these materials are currently commercially available. One reason for that is the insufficient bonding techniques between the TE legs and the hot-side metallic contacts. The current research is focused on the interaction between cobalt-metallized *n*-type 9.104 × 10^−3^ mol % PbI_2_-doped PbTe TE legs and the Ag_0.32_Cu_0.43_In_0.25_ brazing alloy, which is free of volatile species. Clear and fine interfaces without any noticeable formation of adverse brittle intermetallic compounds were observed following prolonged thermal treatment testing. Moreover, a reasonable electrical contact resistance of ~2.25 mΩmm^2^ was observed upon brazing at 600 °C, highlighting the potential of such contacts while developing practical PbTe-based TE generators.

## 1. Introduction

Thermoelectric (TE) devices capable of converting waste heat into useful electricity are being constantly investigated for various applications involving different temperature ranges. *p*-type Bi*_x_*Sb_2-*x*_Te_3_ [1,2,3,4,5,6] and *n*-type Bi_2_Te_3-*x*_Se*_x_* [7,8,9,10,11] alloys are the most commonly investigated for operating temperatures of up to 300 °C, while for temperatures beyond 500 °C, filled skutterudites [12,13], half-Heusler [14], and silicide-based [15] compositions are the main focus. At the intermediate 300–500 °C temperature range, PbTe-based compositions are the most thermoelectrically efficient [16,17,18,19,20,21,22,23,24,25,26,27,28,29,30,31,32,33], where a very interesting, highly explored composition is the *n*-type 9.104 × 10^−3^ mol % PbI_2_-doped PbTe [34,35,36,37,38].

Regardless of the great importance for increasing technology readiness, few commercial TE devices capable of efficient operation at intermediate to high temperatures are available to date (e.g., by TECTEG, Thermo-Gen, Tellurex, and Gentherm Inc.). So far there have only been a few reports dealing with details of fabrication of highly efficient PbTe-based devices [39]. A possible reason for that can be insufficient contact-bonding techniques, resulting in high-contact resistances and overall instability in long-term use. Although NASA has employed TE devices for deep space missions, such as the Multi-Mission Radioisotope Thermoelectric Generator (MMRTG) based on *n*-type PbTe and *p*-type PbSnTe [40,41,42], alternatives are still required for developing highly efficient devices by deep investigation in this field.

In TE couples for power generation applications, *p*- and *n*-type TE elements are usually connected electrically in series by brazing into a conducting strip. For most of the TE materials, direct brazing onto the metallic interconnect is difficult either due to poor wettability, or due to a strong reaction between the brazing and the TE materials, degrading their performance at the working temperature. As an example of the latter, widely used contact materials contain Sn, which reacts with PbTe-based TE elements to create SnTe, which ultimately harms the reliability of the TE device [38].

For these reasons, a metallized contact layer between the TE elements and the brazing material is frequently required. For such contact layers, materials with high thermal and electrical conductivities are favorable with respect to device performance.

**Figure 1:**
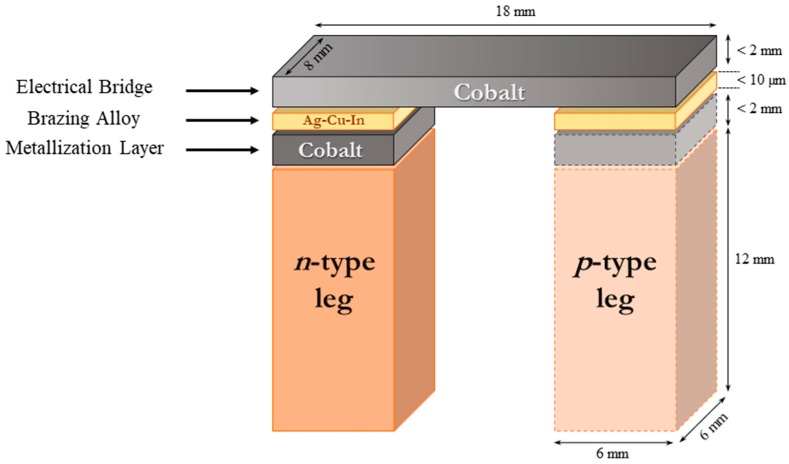
Schematic description of TE couple configuration and stacking layers (and their dimensions) between the TE legs and the hot-side electrical bridge.

To simplify, the above concept is illustrated in the scheme shown in Figure 1. One should note that, as a first step, only the *n*-type TE leg bonding was investigated in the current research, while the *p*-type side, shown schematically for clarifying the proposed concept, is not further discussed in this paper’s framework and will be further investigated in a later paper.

Obtaining sound contacts also requires similar coefficients of thermal expansion (CTE) for the contact layer and the involved TE materials. A system with a significant CTE mismatch will experience mechanical instability, leading to physical, electrical, and thermal disconnection at the vicinity of the contacts. Due to the fact that the TE elements are electrically serially connected in a TE generator, as described above, any single disconnection between the TE legs and the metallic contacts will disable functioning of the entire device.

Relatively thick contact layers at low cost can be joined to the TE elements by hot pressing. Cobalt contact layers have not been widely explored as metallic bridges in PbTe couples. Cobalt has a CTE relatively close to that of the PbTe, featuring ~15 × 10^−6^ K^−1^ and ~21 × 10^−6^ K^−1^, respectively [43], and a low miscibility with Pb and Te [44], eliminating adverse interaction layers that may increase thermal and contact resistances, and therefore might be considered as a reliable contact layer with this class of TE materials. It is worth mentioning that iron was also considered a candidate to serve as a contact layer. However, using iron is challenging due to the fact that it is subjected to more rapid oxidation [45], and a buffer layer is required in order to overcome CTE mismatch [46]. These two facts complicate the brazing process prior to and during brazing.

As for the brazing alloy, the eutectic Ag_0.32_Cu_0.43_In_0.25_, which has not been widely investigated in the TE field, has a melting point of ~564 °C, which is suitable for prolonged operation of PbTe-based TE generators at a maximal temperature of 500 °C from one side, but sufficiently low (<600 °C) to avoid deterioration of the TE elements [47,48] and to minimize any diffusion between the layers during the brazing process on the other side. In addition, it does not contain commonly used elements, such as Zn and Cd, which are highly volatile at working temperatures (<500 °C).

Brazing materials also need to exhibit high wetting capability with the contact layers, but low reactivity to avoid formation of mechanically brittle intermetallic compounds, in addition to low diffusivity through the contact layers to avoid poisoning of the TE materials and thereby affecting the electronic properties. These three properties should be investigated in detail, prior to applying any specific brazing materials for TE power generation applications.

In terms of cost-effectiveness, an Ag–Cu–In-based composition might be considered expensive, but taking into account the low quantity used (<2 g per couple, equivalent to less than $2), the composition is still relevant.

In the current research, the compatibility between cobalt-metallized *n*-type PbI_2_-doped PbTe TE legs and an Ag_0.32_Cu_0.43_In_0.25_ brazing alloy was investigated as our first step for developing a TE PbTe-based power generation device, capable of operating at temperatures up to 500 °C (Figure 1).

## 2. Experimental

*n*-type 9.104 × 10^−3^ mol % PbI_2_-doped PbTe was synthesized from pure elements (purity of 5 N) in evacuated quartz ampoules under vacuum of 10^−6^ Torr, in a rocking furnace (Thermcraft Inc., Winston Salem, NC, USA) at 1000 °C/15 min, then water quenched. The cast ingots were milled to a maximal powder particle size of ~200 µm using agate mortar and pestle.

Cobalt discs (Ø30 mm × 2 mm) were prepared by hot-pressing a pure elemental (5 N) powder under a mechanical pressure of 35 MPa at 850 °C for 30 min. It is noteworthy that the thickness of the cobalt plates can be further adjusted by polishing to minimum thickness of a few microns, where it still functions as an effective diffusion barrier.

Co-metallization on both sides of the PbTe legs (Co–PbTe–Co) was obtained by simultaneously hot pressing (HPW5 Hot Press, FCT System GmbH, Rauenstein, Germany) the two previously hot-pressed cobalt discs with PbTe sieved powder under a mechanical pressure of 20 MPa at 720 °C for 30 min, conditions used for PbTe sintering. Prior to pressing, the cobalt discs were coarsely polished on one side and finely polished on the other, while the coarsely polished side of the cobalt discs faced the PbTe powder for better adhesion. All of the above hot-pressing stages resulted in high density values of >98% of the theoretical density.

The brazing composition of Ag_0.32_Cu_0.43_In_0.25_ was synthesized via arc-melting (MAM-1; Edmund Bühler, Hechingen, Germany) from pure (5 N) silver, copper, and indium elements under Ar atmosphere, with more than five flipping and re-melting stages to ensure homogeneity. The phase transition temperatures of the brazing alloy were measured using differential scanning calorimetry (STA 449, Netzsch).

The TE transport properties of the cobalt contact layer, *n*-type TE element, and the brazing alloy were measured as follows. The Seebeck coefficient and electrical resistivity were measured by Linseis LSR-3/800 Seebeck coefficient/electrical resistance measuring system. The thermal conductivity was determined using the flash diffusivity method (LFA 457, Netzsch).

The CTE of the cobalt contact layer and the *n*-type PbTe TE element were determined by thermomechanical analysis (TMA 402 F3, Netzsch).

The Co–PbTe–Co array was thermally treated at 520 °C for up to 1000 h; at every 100 h, the electrical resistance was measured by a four-point probe method, and the contact resistance was calculated.

Wetting experiments of the brazing alloy on the cobalt plates were performed as follows. At the first stage, hot-pressed cobalt was sliced into ~2 × 8 × 18 mm^3^ plates, which were coarsely polished and ultrasonically cleaned with acetone. Second, the brazing alloy was ground and applied on the metallic plates, then placed in a furnace at 600, 620, and 650 °C for 5, 15, and 30 min, under a 10% H_2_/90% Ar atmosphere. The contact angles were measured by magnified cross-section optical microscopy images of the brazing material drop on the cobalt plate. Micro-scale characterization of the involved interfaces was conducted using a scanning electron microscope (SEM, JSM-5600, JEOL, Akishima, Japan) equipped with an energy-dispersive X-ray spectroscopy (EDS) detector in order to closely examine the compatibility of Ag_0.32_Cu_0.43_In_0.25_ and the metallic plates.

In order to investigate the long-term stability of the contacts, the Ag_0.32_Cu_0.43_In_0.25_ brazing of two cobalt 8 × 18 mm^2^ plates together at 600 °C for 30 min was performed by applying <2 g of brazing powder between two similar cobalt plates, using a 200 g load. This was followed by thermal treatment at 520 °C (~20 °C above the maximal working temperature) for 1000 h and at 550 °C (~14 °C below the melting point of the brazing material) for 50 h.

## 3. Results and Discussion

### 3.1. Synthesized Materials

**Figure 2:**
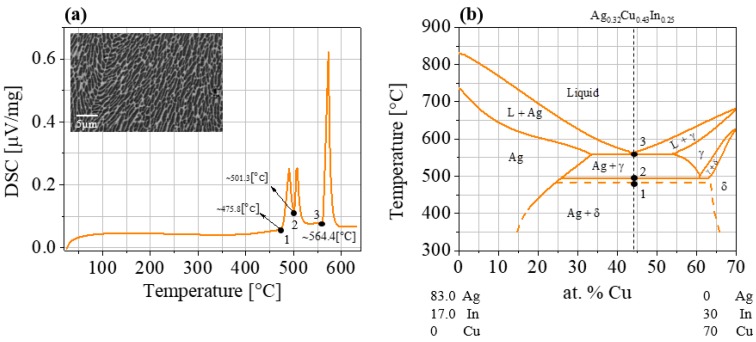
(**a**) Differential scanning calorimetry (DSC) results of the Ag_0.32_Cu_0.43_In_0.25_ brazing alloy. The inset shows an SEM macroscopic view of the obtained eutectic morphology. (**b**) Ag–Cu–In phase diagram of the Ag_0.83_In_0.17_–Cu_7_In_3_ section [49] indicating the two phase transitions (1, 2) and the solid-state eutectic temperature (3) for the currently investigated Ag_0.32_Cu_0.43_In_0.25_ composition.

Following synthesis of the Ag_0.32_Cu_0.43_In_0.25_ brazing alloy, the differential scanning calorimetry results, shown in Figure 2a, indicate three endothermic peaks at ~475 °C, ~501 °C, and ~564 °C. The first two correspond to solid-state phase transitions and the latter reflects the eutectic temperature. These results are in good agreement with the temperatures associated with this composition in the Ag–Cu–In phase diagram in Figure 2b [49]. The horizontal dashed line at ~475 °C corresponds to the ternary peritectic triple point between the Ag, γ-Cu_7_In_3_, and ζ-Ag_3_In phases.

The microstructure of the brazing alloy, observed with SEM (inset of Figure 2a), clearly indicates a eutectic morphology, as was expected (see phase diagram, Figure 2b). As analyzed by EDS, the bright phase was identified as the Ag-rich phase and the dark phase as the γ-Cu_7_In_3_ phase.

It can be easily understood from the phase diagram that the currently investigated Ag_0.32_Cu_0.43_In_0.25_ brazing composition was chosen as one exhibiting a melting point (Point 3) slightly higher than the maximal working temperature of PbTe-based TE devices (~500 °C) but lower than the deterioration temperature (~600 °C [48]) of the TE elements.

**Figure 3:**
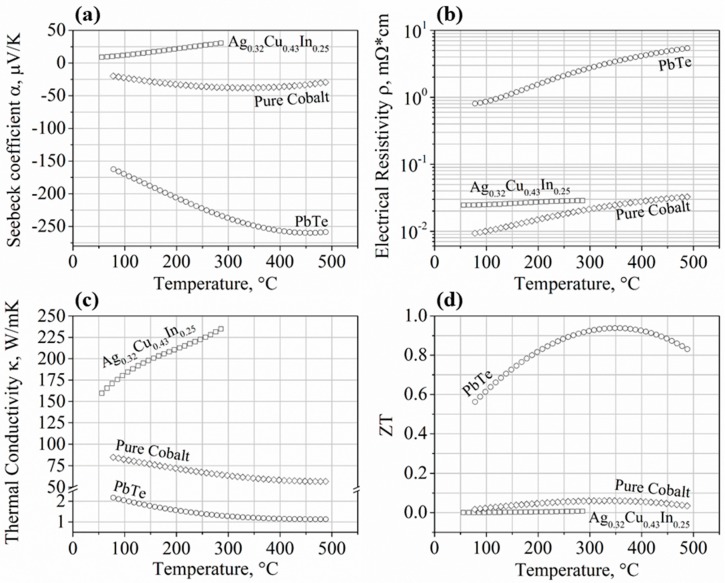
Temperature dependence of the TE properties of the Ag_0.32_Cu_0.43_In_0.25_ brazing alloy, cobalt contact layer, and *n*-type PbTe following hot pressing; (**a**) Seebeck coefficient, *α*; (**b**) electrical resistivity, *ρ*, plotted on a logarithmic scale; (**c**) thermal conductivity, *κ*; (**d**) the dimensionless figure of merit, *ZT* (=*α*^2^T/*ρ*/*κ*, where *T* is absolute temperature).

The temperature dependencies of the TE transport properties of the investigated cobalt contact layer, the *n*-type PbTe element, and the Ag_0.32_Cu_0.43_In_0.25_ brazing alloy, are presented in Figure 3a–d.

It can be clearly seen in Figure 3a that, as expected, the PbTe TE alloy exhibits a negative Seebeck coefficient with high absolute values, indicating its *n*-type conduction nature and high TE potential.

The metallic nature of the Ag_0.32_Cu_0.43_In_0.25_ brazing alloy and the cobalt contact layer, compared to the semiconducting nature of the TE element, can be observed by the low *α* and *ρ* (Figure 3a,b, respectively) and the high *κ* (Figure 3c) values of these compositions. These results clearly indicate the high potential of the brazing material and the cobalt contact layers to exhibit very low electrical and thermal contact resistances while being applied in TE devices. It is worth mentioning that these properties of the brazing alloy and the cobalt are an important prerequisite, but not the only ones. The main factors to be considered are the contact resistances between the three materials that might increase the internal resistance of a module, resulting in decreased generator efficiency. Thus, further interface analyses as well as the interfaces’ thermal stability are shown below.

Concerning the *ZT* (Figure 3d), negligible values were obtained for both the brazing alloy and the cobalt, indicating that they are not expected to contribute to the TE efficiency due to their metallic nature, exhibiting a significantly higher carrier concentration than optimal for the TE applications.

Those transport properties of the brazing alloy and the cobalt are brought as a reference in favor of more accurate future modeling.

### 3.2. Thermoelectric Element-Cobalt Interface

**Figure 4:**
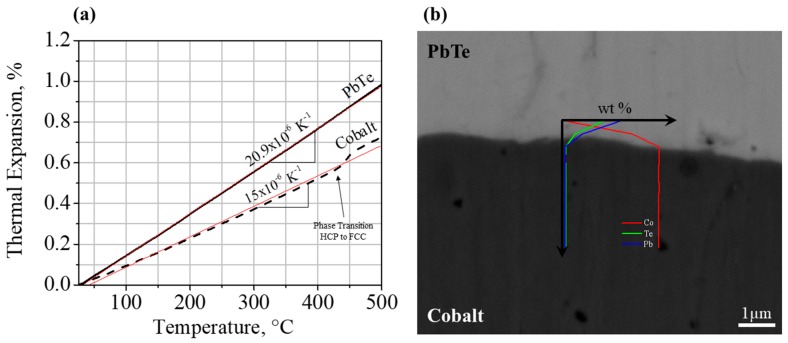

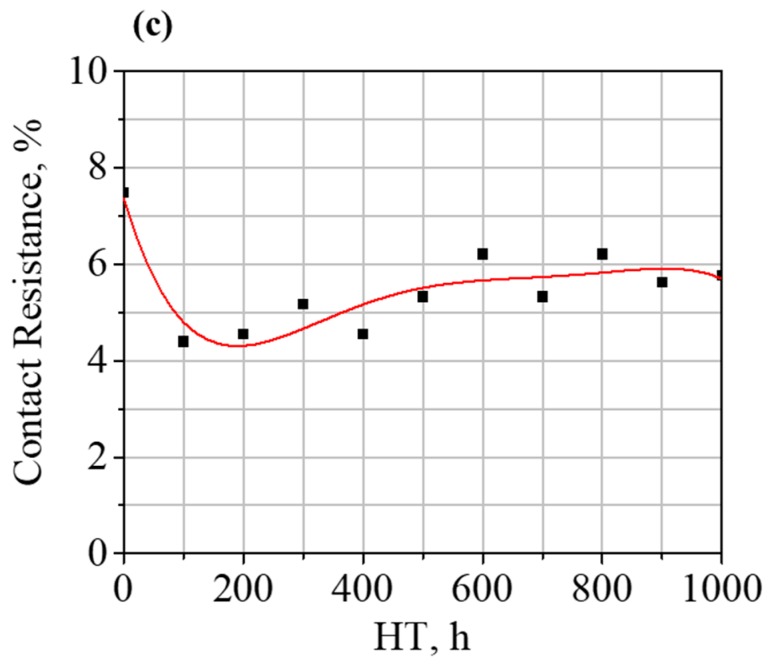
(**a**) Thermomechanical analysis results of the investigated *n*-type PbTe and pure cobalt; (**b**) SEM micrograph of the cobalt–PbTe interface following 520 °C thermal treatments after 1000 h, and (**c**) electrical contact resistance percentage of the cobalt–PbTe interface following 520 °C thermal treatments.

The coefficient of thermal expansion (CTE) values of the cobalt and the *n*-type PbTe TE element, as measured using thermomechanical analysis (Figure 4a), were found to be 15 × 10^−6^ K^−1^ and 20.9 × 10^−6^ K^−1^, respectively. Therefore, no adverse mechanical effects due to CTE mismatch are expected during the operation of the TE device at the expected operation conditions.

Following 1000 h of thermal treatments at 520 °C of the Co–PbTe–Co array, continuous and sound interfaces between the layers were observed, as shown in Figure 4b.

The contact resistance of Co–PbTe interface, *R*_Co–PbTe_, was determined using Equation (1):

(1) $$R_{4\;\mathrm{point}}=R_{\mathrm{PbTe}}+R_{\mathrm{Co}}+2\cdot R_{\mathrm{Co}-\mathrm{PbTe}}=\frac{\rho_{\mathrm{PbTe}}\cdot l_{\mathrm{PbTe}}}{A_{\mathrm{PbTe}}}+\frac{\rho_{\mathrm{Co}}\cdot l_{\mathrm{Co}}}{A_{\mathrm{Co}}}+\;2\cdot R_{\mathrm{Co}-\mathrm{PbTe}}$$

where *R*_4point_ is the measured electrical resistance of the Co–PbTe–Co array after bonding, *R*_PbTe_ is the measured electrical resistance of the *n*-type TE leg prior bonding, and *R*_Co_ is the electrical resistance of the cobalt and considered negligible.

*R*_Co–PbTe_ was found to be ~0.1 mΩ (~3 mΩmm^2^). In earlier studies, a contact resistance value of less than ~10 mΩmm^2^ has been mentioned as desirable for thermoelectric applications [50]. Our results are of the same order of magnitude compared to reported contacts using other metals, such as Ni– and Fe–PbTe joints [45,51].

Following thermal treatments at 520 °C, the electrical contact resistance percentage of the cobalt–PbTe interface was slightly changed with every 100 h. Up to 2% change was seen after 1000 h (as shown in Figure 4c), indicating a high contact stability, as required in a prolonged operation of practical TE applications, even at a higher temperature than the expected maximal working temperature of 500 °C. The drop in contact resistance followed by plateau behavior might be related to the fact that the as-bonded interface (before heat treatments) grips by mechanical means (creating more bonding surface by previously coarsely polishing the cobalt plates), while after heat treatments, chemical bonding by diffusion between the materials might occur (diffusion that we cannot further indicate using SEM/EDS analysis due to low resolution), making the bond even stronger by chemical means without relying solely on mechanical bonding.

The fact that there was no intermediate layer at the interface makes the cobalt more favorable for PbTe-based TE devices than the other metals mentioned above. Since the analytical resolution of a conventional EDS analysis is at best 1 µm, the exact diffusion lengths of these elements could not be identified.

For assuring there were no electrical effects of cobalt as a dopant on the transport properties of PbTe after the thermal treatments, the room temperature of both the Seebeck coefficient and the resistivity of the PbTe leg was re-measured and found to be within ±5% measurement error to the original results, prior to the thermal treatments.

### 3.3. Cobalt–Brazing Alloy Interface

**Figure 5:**
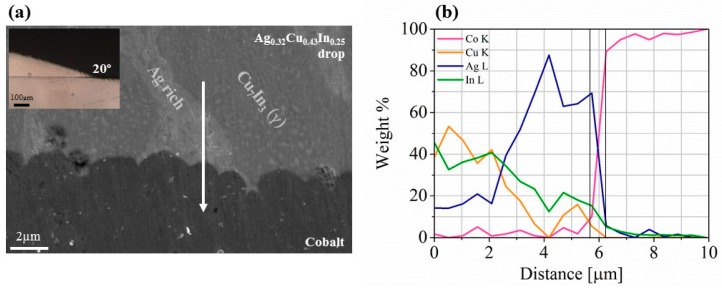
(**a**) SEM micrograph and (**b**) EDS line-scan across the cobalt–Ag_0.32_Cu_0.43_In_0.25_ brazing alloy interface following wettability test at 600 °C for 30 min. The inset in (**a**) indicates an optical microscope view of the low dihedral angle.

As explained above, wettability tests between the Ag_0.32_Cu_0.43_In_0.25_ brazing alloy and the cobalt contact layers were performed at 600 °C, 620 °C, and 650 °C for 5, 15, and 30 min. As expected, the wetting angle between the brazing alloy and the metallic contacts varied as a function of the applied temperature and time. At 600 °C, non-continuous and partially disconnected interface was observed following the first 5 min, a high non-wetting contact angle was observed during the next 10 min, but a significant improvement with a low dihedral angle of ~20° was observed after 30 min in total, as can be seen in the inset of Figure 5.

Upon raising the temperature to 620 °C and 650 °C for 15 min, a slight improvement was observed showing dihedral angles of ~30° and ~15°, respectively. At the highest temperature of 650 °C, an equilibrium contact angle of ~15° was obtained after the first 5 min.

Due to the requirement of lowering the brazing temperature as much as possible, mainly to minimize diffusion between the brazing alloy and cobalt, the optimal brazing condition maintaining adequate wetting of the cobalt layers by the brazing material was chosen as 600 °C/30 min, enabling sound and continuous interfaces. At this condition, a reasonable electrical contact resistance of ~2.25 mΩmm^2^ was measured, highlighting the potential of such interfaces for being incorporated in practical TE devices [50].

**Figure 6:**
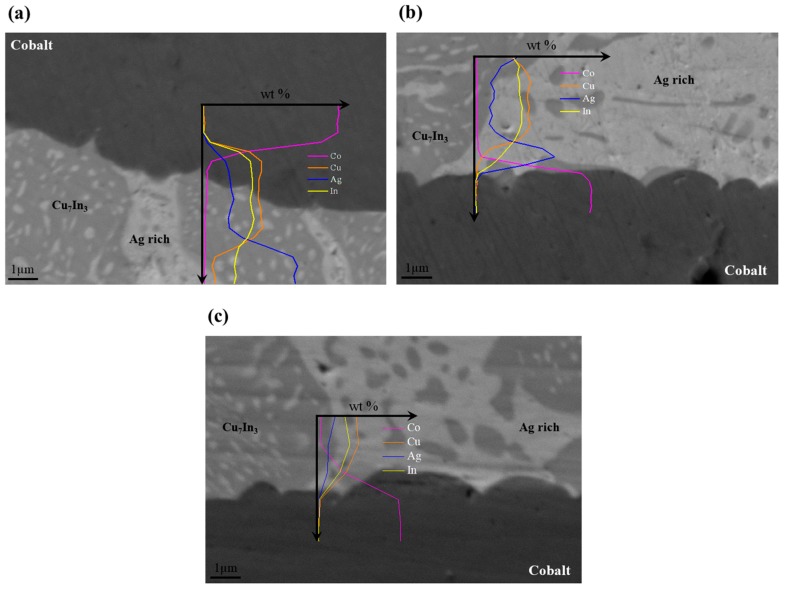
SEM micrographs and EDS line-scans following Ag_0.32_Cu_0.43_In_0.25_ brazing of similar cobalt plates to each other (**a**) before heat treatment (HT); (**b**) after HT at 550 °C for 50 h; and (**c**) after HT at 520 °C for 1000 h.

The stability of the cobalt–brazing alloy interface was analyzed for two similar cobalt plates brazed together by Ag_0.32_Cu_0.43_In_0.25_ under the above-specified optimal brazing conditions (600 °C/30 min). The interface was analyzed following 1000 h of thermal treating at 520 °C, demonstrating the maximal possible working temperature. In addition, it was analyzed following thermal treating at 550 °C, demonstrating severe short-term conditions (50 h) at 14 °C lower than the brazing alloy’s melting temperature. The interfaces between the cobalt plates and the Ag_0.32_Cu_0.43_In_0.25_ alloy remained smooth and clear without any evidence of a noticeable interaction layer evolvement, as can be seen when comparing Figure 6b,c to the as-brazed condition (Figure 6a). It is evident by the micrographs that, under the applied conditions, limited diffusion of elemental Ag, Cu, and In from the brazing alloy into the cobalt plates is apparent, highlighting the high potential of the applied bonding method to be incorporated in long-term TE applications. As mentioned, since the analytical resolution of a conventional EDS analysis is at best 1 µm, the exact diffusion lengths of these elements in cobalt could not be identified, but it can be concluded that cobalt thickness needs to be of the order of a few microns in order to function as a diffusion barrier.

It is worth mentioning that these thermal treatment results (Figure 6b,c) verify that the phase transition, which is in the solid state as indicated in Figure 2, is not expected to adversely affect the stability of the couples at temperatures above the phase transitions temperature. Moreover, the fact that this brazing composition is very ductile, especially at operating temperatures, enables some compensation of any CTE mismatch. The thermomechanical stability of the joints does not seem to be affected even after long-term operation.

## 4. Conclusions

For increasing the technology readiness level (TRL) of TE converters, based on the widely investigated highly efficient PbTe compound, the current research focused on bonding *n*-type 9.104 × 10^−3^ mol % PbI_2_-doped PbTe to cobalt metallic contacts, as potential hot- and cold-side electrical bridges of a TE couple operating at temperatures up to 500 °C. For this purpose, a procedure for cobalt metallization of the investigated *n*-type PbTe compound is being reported as the first stage for obtaining such couples. The metallization layer exhibits high compatibility in terms of CTE matching and minimal chemical interaction, preventing potential degradation of the TE transport properties under the expected operation conditions. A promising solution would be a very thin layer (few microns) of cobalt where it functions as an effective diffusion barrier.

At the second stage, the Ag_0.32_Cu_0.43_In_0.25_ alloy was investigated as a brazing composition between the metallized cobalt layers of the TE compound and cobalt hot/cold side electrical bridges of a PbTe-based TE couple.

The stability of the Co–PbTe–Co contacts following metallization (also in terms of electrical contact resistances) following prolonged thermal treatments at temperatures higher than the maximal expected operation temperature (500 °C) showed no evidence of interface deterioration.

Following Ag_0.32_Cu_0.43_In_0.25_ brazing at 600 °C, smooth and clear interfaces with a reasonable electrical resistance of ~2.25 mΩmm^2^ were obtained between the cobalt layers and the brazing alloy, without any noticeable formation of adverse intermetallic compounds, even after thermal treatments above the expected maximal working temperature.

The final stage toward a highly efficient TE generator is to build a prototype to work in a lab testing facility. These reported findings clearly indicate the high potential of the proposed cobalt metallization and brazing procedures for being incorporated in practical TE applications up to 500 °C.
